# Infrared Image Denoising Algorithm Based on Wavelet Transform and Self-Attention Mechanism

**DOI:** 10.3390/s26020523

**Published:** 2026-01-13

**Authors:** Hongmei Li, Yang Zhang, Luxia Yang, Hongrui Zhang

**Affiliations:** College of Computer Science and Technology, Taiyuan Normal University, Jinzhong 030619, China; zhangyangzy1997@163.com (Y.Z.); ylxfyz328@163.com (L.Y.); zhanghongrui@tynu.edu.cn (H.Z.)

**Keywords:** infrared image denoising, transformer, wavelet transform self-attention, multi-scale gated linear unit, parallel hybrid attention

## Abstract

Infrared images are often degraded by complex noise due to hardware and environmental factors, posing challenges for subsequent processing and target detection. To overcome the shortcomings of existing denoising methods in balancing noise removal and detail preservation, this paper proposes a Wavelet Transform Enhanced Infrared Denoising Model (WTEIDM). Firstly, a Wavelet Transform Self-Attention (WTSA) is designed, which combines the frequency-domain decomposition ability of the discrete wavelet transform (DWT) with the dynamic weighting mechanism of self-attention to achieve effective separation of noise and detail. Secondly, a Multi-Scale Gated Linear Unit (MSGLU) is devised to improve the ability to capture detail information and dynamically control features through dual-branch multi-scale depth-wise convolution and gating strategy. Finally, a Parallel Hybrid Attention Module (PHAM) is proposed to enhance cross-dimensional feature fusion effect through the parallel cross-interaction of spatial and channel attention. Extensive experiments are conducted on five infrared datasets under different noise levels (σ = 15, 25, and 50). The results demonstrate that the proposed WTEIDM outperforms several state-of-the-art denoising algorithms on both PSNR and SSIM metrics, confirming its superior generalization capability and robustness.

## 1. Introduction

As a detection technique for sensing invisible thermal radiation, infrared imaging has been widely applied in critical fields such as military reconnaissance, medical diagnosis, industrial monitoring, and security systems, owing to its strong penetration capability in low-light conditions and excellent resistance to environmental interference. However, during the acquisition and transmission processes, infrared images are inevitably corrupted by noise due to environmental factors or the imaging device itself [1]. Common noise types include impulse noise and Gaussian noise, with the latter being particularly destructive and challenging to remove [2]. Such noise not only degrades the visual quality of infrared images but also adversely affects subsequent analysis and processing tasks. Therefore, infrared image denoising holds significant theoretical research value and practical importance.

Given the prevalence and detrimental effects of noise in infrared images, researchers have long been dedicated to developing effective denoising algorithms. Existing approaches can be broadly categorized into two groups: signal processing methods based on image statistics and data-driven deep learning techniques. Traditional signal processing methods construct mathematical models based on statistical characteristics or spatial structural features of images to separate noise from authentic signals. Common techniques approaches include: spatial domain methods (e.g., mean filtering [3], median filtering [4], bilateral filtering [5]), frequency domain methods (e.g., Wiener filtering [6], wavelet transform [7], Fourier transform [8]), and hybrid spatial-frequency combined methods such as non-local means (NLM) [9], block-matching and 3D filtering (BM3D) [10]. Among this series of denoising methods, certain limitations exist in handling Gaussian noise: mean filtering tends to cause blurring of image edges and detail textures; the effectiveness of Wiener filtering relies on signal and noise prior knowledge that is difficult to accurately obtain; while the relatively high-performing BM3D algorithm also heavily depends on manually tuning of predefined parameters such as search window size, patch dimensions, similarity thresholds. Consequently, traditional denoising methods exhibit high dependency on specific model assumptions and manual parameter optimization, making it challenging to adaptively balance denoising strength and the preservation of critical details (e.g., weak edges and textures) when processing complex and variable infrared noise.

In recent years, the rapid advancement of artificial intelligence, particularly the breakthroughs in data-driven deep learning within computer vision, has provided novel technical pathways for infrared image denoising research. Unlike traditional methods that rely on handcrafted models and features, the denoising approaches based deep learning leverage the powerful end-to-end learning capability of deep neural networks. These methods can autonomously learn the statistical distribution of noise and the underlying structural information of images directly from large-scale datasets containing noisy images and their corresponding clean versions. Owing to this distinct advantage, this research direction has garnered extensive attention and in-depth investigation from scholars worldwide. Among various deep learning architectures, Transformer model has emerged as a promising candidate for low-level vision tasks due to its ability to effectively model long-range dependencies through self-attention mechanisms, which has also driven preliminary explorations of its application in image denoising. For instance, methods like Pureformer [11] and TBSN [12] have demonstrated promising results in natural image denoising by modeling long-range dependencies through self-attention mechanisms.

However, directly applying the Transformer architectures to infrared images with complex noise still faces multifaceted challenges. First, infrared images generally suffer from low signal-to-noise ratio (SNR). As traditional self-attention operates globally in pixel space, it often struggles to distinguish noise from genuine details, thereby resulting in over-smoothing or residual noise. Second, most vision Transformers lack explicit modeling of frequency domain information, which limits their ability to effectively capture the distinct distribution of noise and structural details across different frequency bands. Furthermore, the standard Feedforward Network (FFN) in Transformers is typically composed of fully connected layers, which have limited ability to adapt to local spatial structures and multi-scale features. This limitation hinders the preservation of critical details such as edges and textures. Multi-scale feature processing has been widely validated as an effective strategy in low-level vision tasks, enhancing scale-aware representation learning and strengthening feature interaction across receptive fields. Jin et al. [13] proposed a multi-branch Taylor expansion-based convolution framework, demonstrating strong capability in scale-aware feature interaction for image restoration. DDMSNet [14] designed a dual-branch dynamic multi-scale module that adjusts receptive fields according to input content, providing a valuable reference for multi-scale feature extraction in denoising tasks. However, directly adopting such multi-scale designs may still be insufficient for infrared denoising. Due to the low SNR, weak edges and textures are easily overwhelmed by noise, demanding more selective and adaptive feature modulation. These limitations have motivated ongoing efforts to optimize Transformer-based denoising models, while also prompting researchers to explore alternative deep learning frameworks to address infrared image denoising challenges.

Although dedicated Transformer-based models for infrared denoising are still in their early stages, existing deep learning methods—developed based on attention, reinforcement learning, or adversarial learning frameworks—have provided valuable insights into feature extraction and noise suppression. For instance, Li et al. [15] introduced a deep learning model incorporating a second-order attention mechanism and non-local modules. While it demonstrates effective feature extraction and noise fitting, its capability to preserve fine details in infrared image remains limited. Zhang et al. [16] designed a deep reinforcement learning model that optimizes the extraction of star targets under few frames’ conditions. However, this approach tends to cause blurring of details surrounding the targets. Hu et al. [17] proposed a symmetric multi-scale encoder–decoder structure model, which enhances the reconstruction quality of infrared images through multi-scale information extraction guidance. However, some loss of edge details remains. Yang et al. [18] employed adversarial learning combined with a multi-level feature attention network (MLFAN) to achieve effective denoising, but the approach still shows deficiencies in preserving fine textures when integrating features from different levels. The SwinDenoising method proposed by Wu et al. [19] enhanced robustness by capturing both local and global features. However, its ability to restore details within complex backgrounds remains an area for improvement. Although these deep learning approaches have achieved promising denoising results, a key challenge that requires further attention is achieving an optimal balance between denoising strength and the preservation of detail integrity.

To effectively address the aforementioned challenges, this paper proposes a Wavelet Transform enhanced Infrared Image denoising model (WTEIDM). The main contributions are summarized as follows:

(1) The Wavelet Transform Self-Attention (WTSA) mechanism is designed, which leverages wavelet transformation to decompose features and accurately capture critical information. By integrating self-attention, it achieves a balance between infrared denoising and detail preservation.
(2) A Multi-scale Gated Linear Unit (MSGLU) is developed, which employs 3 × 3 and 5 × 5 depth-wise convolutions to construct multi-scale branches. Coupled with a gating mechanism, it enables dynamic feature modulation and enhances the capture of detailed information.
(3) A Parallel Hybrid Attention Module (PHAM) is introduced, which performs feature fusion through the parallel and interactive operation of spatial and channel attention. This enhances cross-dimensional feature integration and improving detail retention capability.

## 2. The Proposed WTEIDM

**Figure 1:**
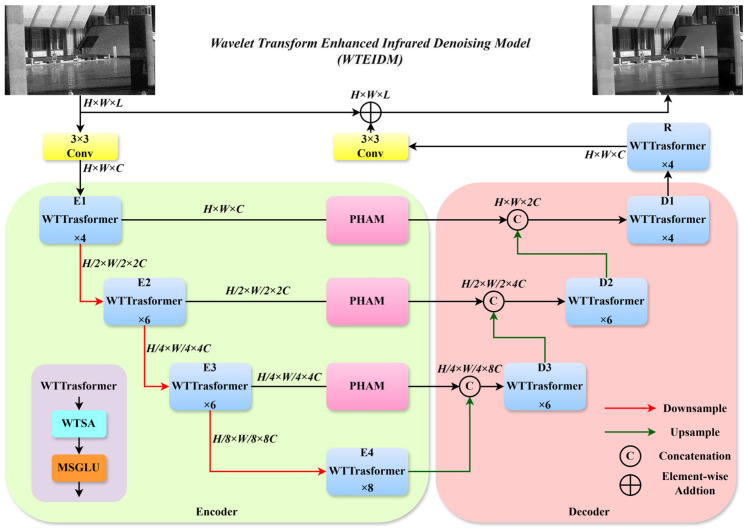
The network structure of WTEIDM. Data flow (solid arrows): Noisy image → Conv/Normalization (initial feature extraction) → Encoder (stacked WTTransformer: WTSA→MSGLU) → PHAM (cross-dimensional fusion) → Decoder (stacked WTTransformer + up-sampling) → Conv (output denoised image); Skip connections (dashed arrows) bridge encoder–decoder features of the same scale to compensate detail loss. Module collaboration: WTSA (frequency-domain noise-detail separation) and MSGLU (multi-scale spatial modulation) form the core of WTTransformer; PHAM (between encoder–decoder) fuses spatial-channel features, forming a “separation-modulation-fusion-reconstruction” closed loop.

To address the challenge of balancing noise suppression and detail preservation in infrared image denoising, this paper proposes a wavelet transform enhanced infrared denoising model (WTEIDM). The model is built upon a U-Net architecture integrated with a Transformer backbone, with its overall structure illustrated in Figure 1. In the encoder part, a WTTransformer module is designed, which integrates a wavelet transform self-attention (WTSA) mechanism and a multi-scale gated linear unit (MSGLU). The WTSA performs feature decomposition via wavelet transformation to accurately capture essential information, while the MSGLU applies multi-scale depth-wise convolution and dynamic gating to enhance the representation of edges, textures, and other detailed structures during noise removal. Between the encoder and decoder, a parallel hybrid attention module (PHAM) is incorporated to perform feature fusion via the synergistic operation of parallel spatial and channel attention pathways. This design enhances the discrimination between noise and meaningful details and reinforces the cross-stage propagation of critical features, thereby mitigating detail loss during denoising. The decoder adopts a layered WTTransformer structure combined with up-sampling and feature concatenation to progressively restore image details. In summary, the proposed model effectively suppresses noise while preserving structural integrity through collaborative encoding, fusion, and decoding, which significantly improves the denoising quality of infrared images.

### 2.1. Wavelet Transform Self-Attention (WTSA)

In infrared image denoising, when the traditional self-attention mechanisms operate in the pixel space for feature interaction, it is susceptible to the low signal-to-noise ratio characteristic of infrared images. Consequently, it struggles to effectively distinguish between noise and detail features. Furthermore, due to its limited ability to discriminate features in the frequency domain, it fails to adequately capture detail information embedded in different frequency components. This often leads to either loss of detail or noise retention in the denoised output.

**Figure 2:**
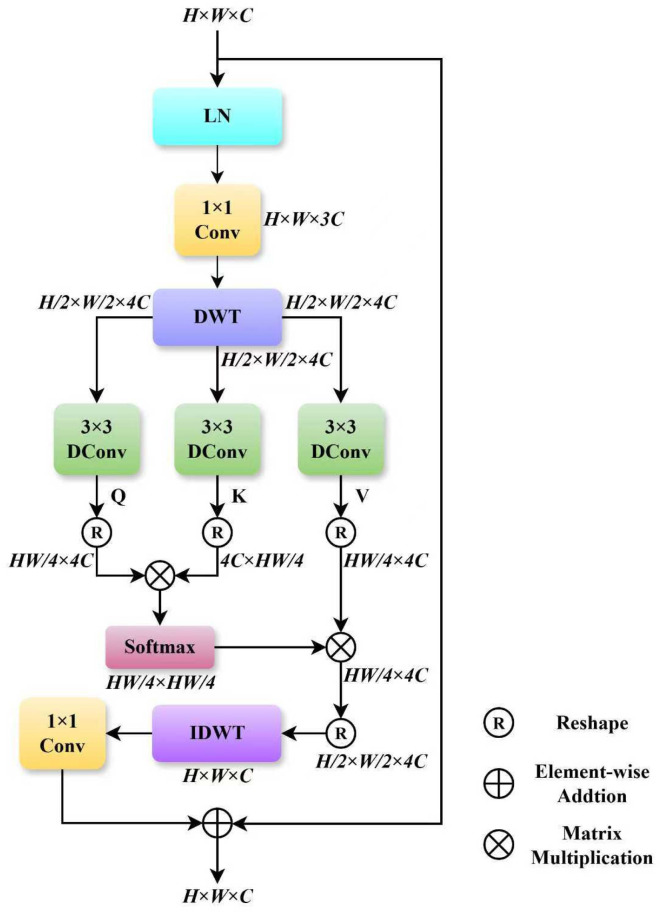
The network structure of WTSA module.

To overcome these shortcomings, we design a wavelet transform self-attention (WTSA) mechanism. This model integrates the frequency-domain decomposition capability of discrete wavelet transform (DWT) with the dynamic feature weighting advantages of self-attention, allowing for accurate separation of noise and meaningful detail features. The structure of WTSA is illustrated in Figure 2.

The core workflow of the WTSA module comprises four stages: feature preprocessing, wavelet frequency-domain decomposition, attentive feature interaction, and feature reconstruction. First, the input feature map $X\in R^{H\times W\times C}$ undergoes layer normalization for distribution standardization, which stabilizes training and suppresses gradient fluctuation. Subsequently, the normalized features are passed through a 1 × 1 convolution to adjust the channel dimension to 3C, yielding an intermediate feature $X_{conv}\;\in R^{H\times W\times 3C}$. This channel transformation step reduces parameter redundancy for the subsequent wavelet decomposition and attention computations.

Next, the feature map $X_{conv}$ is decomposed by the Discrete Wavelet Transform (DWT) into four sub-band features $X_{dwt\;}\in R^{H/2\times W/2\times 4C}$. In this process, the Haar wavelet basis function is adopted, whose step-shaped filter can effectively capture local edge information in the image, suppressing feature redundancy while enhancing inter-channel discriminability. Moreover, the Haar wavelet exhibits high computational efficiency, contributing to a favorable trade-off between feature representation accuracy and computational cost. The four resulting sub bands consist of one low-frequency approximation component, which carries the global structural information of the image, and three high-frequency detail components that encompass detail features such as edges and texture, along with noise. This frequency-domain decomposition achieves a preliminary separation of noise and details in the frequency domain, providing more discriminative feature representations for subsequent attention mechanism. In the stage of attentive feature interaction, $X_{dwt\;}$ is fed into three parallel 3 × 3 depth-wise convolutions (DConv) to extract the *Query* (Q), *Key* (*K*), and *Value* (*V*) feature components, respectively. The formulation is as follows:

(1) $$Q=DConv_{3\times 3}(X_{dwt\;})$$

(2) $$K=DConv_{3\times 3}(X_{dwt\;})$$

(3) $$V=DConv_{3\times 3}(X_{dwt\;})$$

The 3 × 3 depth-wise convolution leverages its local receptive field to enhance the extraction of local features within the four sub-bands, enabling the generated $Q\in R^{H/2\times W/2\times 4C}$, $K\in R^{H/2\times W/2\times 4C}$ and $V\in R^{H/2\times W/2\times 4C}$ features to better align with the data distribution in the wavelet domain. Subsequently, *Q*, *K* and *V* are reshaped into sequence forms $\hat{Q}$, $\hat{K}$ and $\hat{V}$, respectively. The attention weight matrix is computed as follows:

(4) $$\mathrm{Attention}(\hat{Q},\;\hat{K},\;\hat{V})=Softmax(\;\frac{\hat{Q}{\hat{K}}^{T}}{d}\;)\hat{V}$$

where $\hat{Q}\in R^{HW/4\times 4C}$, ${\hat{K}}^{T}\in R^{4C\times HW/4}$, $\hat{V}\in R^{HW/4\times 4C}$, and d denotes a learnable scaling factor, respectively. This process dynamically amplifies informative feature weights while suppressing interference from noise-dominated sub-bands.

Finally, the output of the attention mechanism is reshaped to restore its spatial dimensions, and the four sub-band features are reconstructed via the inverse discrete wavelet transform (IDWT) into $X_{idwt}\;\in R^{H\times W\times C}$. This is followed by a 1 × 1 convolution that optimizes the consistency of the channel features, producing the reconstructed features $X_{rec}\;\in R^{H\times W\times C}$. A residual connection is incorporated to preserve original feature information, and the final output is given by:

(5) $$X_{out}\;=X+X_{rec}\;$$

### 2.2. Multi-Scale Gated Linear Unit (MSGLU)

**Figure 3:**
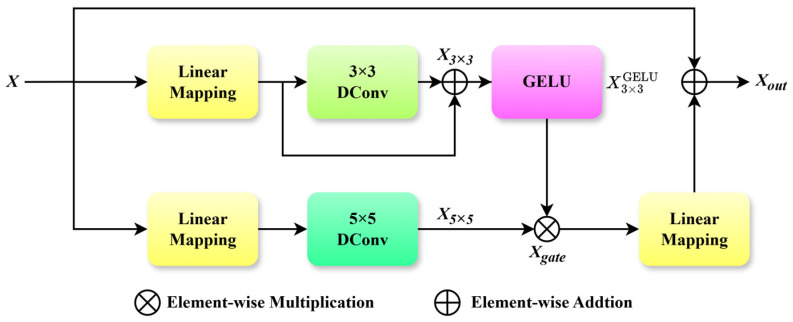
The network structure of MSGLU module.

In the Transformer [20] architecture, the feedforward network (FFN) serves as a critical component following the self-attention layer, enhancing feature representation through bilinear transformation and nonlinear activation. However, in the task of infrared image denoising, the FFN exhibits notable limitations. First, its linear mapping struggles to effectively utilize spatial information and fails to capture inter-pixel correlations, which leads to the loss of edge and texture details. Second, the undifferentiated processing of channels tends to cause noise and meaningful details to overlap, thereby restricting the model’s adaptability to complex noise patterns and multi-scale structures. To overcome these limitations of the FFN, this paper introduces a Multi-scale Gated Linear Unit (MSGLU), as shown in Figure 3. By integrating dual-branch multi-scale depth-wise convolution with a gating mechanism, the MSGLU achieves precise detail feature extraction and dynamic feature modulation.

The MSGLU module adopts a dual-branch parallel architecture. The input feature $X\in R^{H\times W\times C}$ is first processed through linear mapping layers and then fed into two separate branches. In the first branch, the linearly mapped features undergo 3 × 3 depth-wise convolution (DConv) to extract local fine-grained features, such as edges and textures. Subsequently, a residual connection is applied to integrate the original linear features, preserving information completeness and yielding features $X_{3\times 3\;}\in R^{H\times W\times C}$. These features are subsequently activated by a GELU function to enhance nonlinear expressiveness. In the second branch, the linear mapped features generate output features $X_{5\times 5\;}\in R^{H\times W\times C}$ via a 5 × 5 depth-wise convolution (DConv). This operation captures global multi-scale contextual information, including large-scale structural characteristics and noise distribution.

To achieve dynamic modulation of multi-scale features, the MSGLU model incorporates a gating mechanism. This mechanism performs an element-wise multiplication of the GELU-activated features from the 3 × 3 depth-wise convolution branch with those from the 5 × 5 branch, thus adaptively weighting the contributions of features at different scales through gating logic. This process is formulated as:

(6) $$X_{gate\;}=X_{3\times 3}^{GELU}⊗X_{5\times 5}\;$$

where ⊗ denotes element-wise multiplication, and $X_{3\times 3}^{GELU}$ represents the GELU-activated output of the 3 × 3 depth-wise convolution branch.

Finally, the gated features $X_{gate\;}$ undergoes a linear projection to adjust its channels and dimensions, followed by fusion with the original input *X* via a residual connection to produce the final output $X_{out}\in R^{H\times W\times C}$. The expression is as follows:

(7) $$X_{out}\;=X+Linear(X_{gate}\;)$$

### 2.3. Parallel Hybrid Attention Module (PHAM)

Current standalone attention mechanisms, like spatial or channel attention, present clear limitations. Spatial attention effectively captures pixel-position relationships; however, this comes at the cost of ignoring feature variations in the channel dimension. Conversely, while channel attention excels at facilitating information exchange between channels, it often fails to accurately capture localized spatial details.

**Figure 4:**
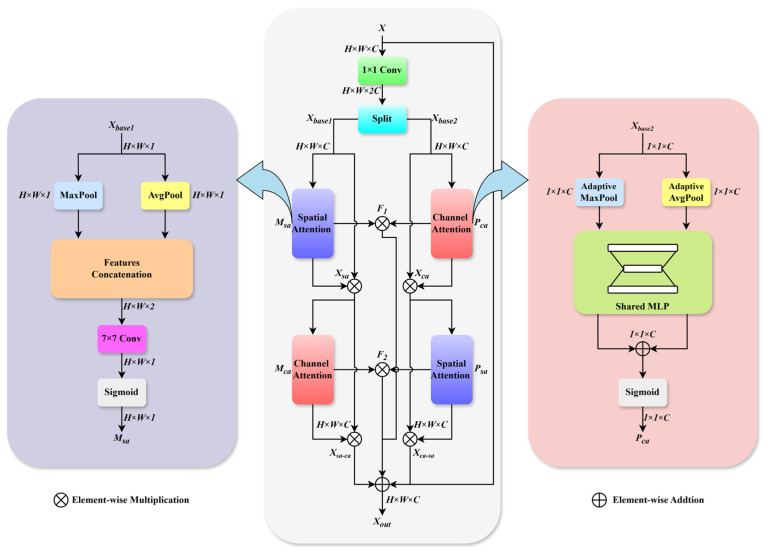
The network structure of PHAM.

To address this issue, we design a parallel hybrid attention module (PHAM), as illustrated in Figure 4. The PHAM consists of two parallel branches, each comprising both spatial and channel attention units. These components are interconnected to enhance the correlations between spatial and channel dimensions, thereby improving noise suppression and detail preservation. Simultaneously, a residual connection is incorporated to preserve the fundamental features and mitigate detail loss during infrared image denoising.

As illustrated in Figure 4, the input feature map $X\in R^{H\times W\times C}$ is first processed via a 1 × 1 convolution to adjust the channel dimension to 2C, producing base features suitable for attention mechanisms. These features are then split into two parallel branches $X_{base1}$ and $X_{base2}$ for dual-attention interaction. The module employs two distinct pathways: a spatially guided channel attention path and a channel-guided spatial attention path. The former prioritizes preserving spatial structural information, while the latter focuses on highlighting inter-channel feature differences, thereby generating distinct intermediate feature representations. Finally, through a cross-fusion operation, the two branches complement each other’s information, achieving a deep enhancement of both spatial and channel features.

In the spatially guided channel attention path, branch features $X_{base1}$ undergoes max pooling and average pooling, respectively, to aggregate spatial information. The pooled features are then concatenated and processed through a 7 × 7 convolution, followed by a sigmoid activation to generate the spatial attention weights $M_{sa}\;\in R^{H\times W\times 1}$. The above process can be expressed by the following formulas:

(8) $$M_{sa}\;=Sigmoid(Conv_{7\times 7}\;(Cat(MaxPool(X_{base1}\;),AvgPool(X_{base1}\;))))$$

These weights $M_{sa}\;$ are applied to the input features via element-wise multiplication to produce spatially enhanced features $X_{sa}\;\in R^{H\times W\times C}$:

(9) $$X_{sa}=X_{base1}⊗M_{sa}\;$$

The spatially enhanced features $X_{sa}\;$then enter a channel attention module, which employs adaptive max pooling (AMP) and adaptive average pooling (AAP), followed by a shared multilayer perceptron (MLP). The MLP outputs are summed and activated by sigmoid to generate channel attention weights $M_{ca}\in R^{1\times 1\times C}$:

(10) $$M_{ca}=Sigmoid(MLP\;(AMP(X_{sa}\;))+MLP\;(AAP(X_{sa}\;)))$$

The final output $X_{ca-sa\;}\in R^{H\times W\times C}$ of this path is obtained by the element-wise multiplication of $X_{sa}\;$ and $M_{sa}\;$:

(11) $$X_{sa-ca\;}=X_{sa}⊗M_{ca}$$

In the complementary channel-guided spatial attention path, branch features $X_{base2}$ are first processed by channel attention to generate weights $P_{ca}\;\in R^{1\times 1\times C}$, which are applied to obtain channel-enhanced features $X_{ca}\in R^{H\times W\times C}$. The above process can be expressed by the following formulas:

(12) $$P_{ca}=Sigmoid(MLP\;(AMP(X_{base2}\;))+MLP\;(AAP(X_{base2}\;)))$$

(13) $$X_{ca}=X_{base2}⊗P_{ca}\;$$

These features $X_{ca}\;$ first undergo spatial attention processing to generate weights $P_{sa}\in R^{H\times W\times 1}$. The final output $X_{ca-sa\;}\in R^{H\times W\times C}$ is then obtained via the element-wise multiplication of $X_{ca}\;$ and $P_{sa}\;$, formulated as follows:

(14) $$P_{sa}\;=Sigmoid(Conv_{7\times 7}\;(Cat(MaxPool(X_{ca}\;),AvgPool(X_{ca}\;))))$$

(15) $$X_{ca-sa\;}=X_{ca}⊗P_{sa}$$

The outputs from both pathways are element-wise summed and further enriched through cross-feature interactions. Specifically, hybrid features $F_{1}\in R^{H\times W\times C}$ and $F_{2}\in R^{H\times W\times C}$ are generated to capture complementary attention patterns:

(16) $$F_{1}=M_{sa}⊗P_{ca}\;$$

(17) $$F_{2}=M_{ca}⊗P_{sa}\;$$

The final output features are obtained by fusing the original input X, the output $X_{ca-sa\;}$ of the spatially guided channel attention module, the output $X_{ca-sa\;}$ of the channel-guided spatial attention module, and the two hybrid features F_1_ and F_2_ through residual connections:

(18) $$X_{out\;}=X+X_{sa-ca\;}+X_{ca-sa}\;+F_{1}+F_{2}$$

## 3. Experiments and Results

### 3.1. Dataset

The experiments employ the public dataset IR700 [21], acquired using a long-wave infrared telescope. The dataset contains a variety of subject images such as people, vehicles, roads, and buildings. It has high resolution and relatively clear content features to ensure the effectiveness of the experiment. The dataset is randomly divided into two subsets: training set (600 images) and testing set (100 images).

To verify the generalization performance of the model, this paper uses four infrared image datasets covering different scenes as an extended test set, namely IR100 [22], Flir [23], ESPOL FIR [24], and DLS-NUC-100 [25], where each dataset contains 100, 50, 101, and 100 images.

### 3.2. Experimental Settings

To facilitate comparison with existing image denoising methods, this study uses simulated infrared noisy images during training and testing. Gaussian noise with a mean of 0 and standard deviations σ of 15, 25, and 50 is added to the original images to simulate infrared noise of varying intensities. By training and testing the comparative models under these noise levels, we comprehensively evaluate their robustness under different noise conditions and ensure comparability of the experimental results. All methods were trained under the same strategy and run independently three times using three different random seeds to assess the stability of the results. The final performance metric was calculated based on the mean and standard deviation of the three runs, and statistical analysis was performed to assess the differences in results using a significance test (*p*-value). When calculating the *p*-value, we used a two-tailed *t*-test, setting the significance level α = 0.05 and the degrees of freedom df = 2.

The WTEIDM adopts a 4-layer encoder–decoder architecture. The four WTTransformer modules (E1, E2, E3, E4) in the encoder are set to 4, 6, 6, and 8 blocks, respectively. The three WTTransformer modules in the decoder (D1, D2, D3) are set to 4, 6, and 6 blocks, respectively. The WTTransformer module R in the final feature refinement stage is set to 4 blocks. Within each WTSA layer, the number of heads in the multi-head self-attention mechanism is set to 1, 2, 4, and 8, respectively, and the head count in module R is set to 1.

During training, image patches of size 64 × 64 pixels are randomly cropped from the training set and trained in mini-batches of 8 samples each. Furthermore, image augmentation is performed via horizontal flipping and random rotations of 90°, 180°, and 270°. The Charbonnier loss function is optimized using the Adam optimizer. The batch size is set to 32, and the training epochs are 3000.

The experiments are conducted on a Windows 11 system equipped with an NVIDIA GeForce RTX 4090 GPU (24 GB VRAM) and an Intel Core i9-13900K CPU @ 3.00 GHz. The software environment is built on Python 3.9, PyTorch 2.0.0, and CUDA 11.8.

### 3.3. Evaluation Indicators

To validate and evaluate the performance of the model, this study adopts two evaluation indicators widely used in the field of image denoising: peak signal-to-noise ratio (*PSNR*) and structural similarity index (*SSIM*).

*PSNR* measures the similarity between an image and its processed version by calculating the ratio of the maximum grayscale value to the mean squared error (MSE) between the original and processed images. A higher PSNR value indicates that the quality of the denoised image is closer to the original image. The formula is as follows:

(19) $$MSE=\frac{1}{m\times n}\;\sum_{i=0}^{m-1}\sum_{j=0}^{n-1}{\left[I(i,j)-I^{\prime }(i,j)\right]}^{2}\;$$

(20) $$PSNR=10\cdot lg(\frac{Max^{2}}{MSE}\;)$$

where *m* and *n* represent the number of rows and columns of the image, *I* denotes the original image, and *I′* represents the denoised image. *MAX* represents the maximum grayscale value of the image.

*SSIM* measures the spatial structural similarity of images by comparing information such as brightness, contrast, and structure in local areas of the images. Its value ranges from 0 to 1, with values closer to 1 indicating greater similarity between the two images. The calculation formula is as follows:

(21) $$SSIM=\frac{\left(2\mu_{x}\;\mu_{y}\;+c_{1}\;)(\sigma_{xy}\;+c_{2}\;\right)}{\left(\mu_{x}^{2}\;+\mu_{y}^{2}\;+c_{1}\;)(\sigma_{x}^{2}\;+\sigma_{y}^{2}\;+c_{2}\;\right)}\;$$

where $\mu_{x}$ and $\mu_{y}$ represent the average of the original and the denoised images respectively. $\sigma_{x}^{2}$ and $\sigma_{y}^{2}$ denote their variances, $\sigma_{xy}$ is the covariance between them, and $c_{1}$, $c_{2}$ are constants to stabilize the division.

### 3.4. Ablation Experiments

To validate the contribution of each module in the WTEIDM to infrared image denoising performance, we adopt a traditional Transformer architecture as the baseline (BASIC) and train it on the IR700 dataset with Gaussian noise at σ = 15. Experiments are conducted on five test sets: IR700_test, IR100, Flir, ESPOL FIR, and DLS-NUC-100. The statistical significance of the results was assessed by calculating *p*-values in comparison with the BASIC baseline, and the detailed results are summarized in Table 1.

The experimental results show that the introduction of the WTSA module yields improvements of approximately 0.09 dB in PSNR and 0.0025 in SSIM, with gains being statistically significant (*p* < 0.05) across all test sets. This indicates that the module exhibits significant effectiveness in noise reduction and feature preservation. Further addition of the MSGLU module leads to more significant performance gains, with PSNR increasing by an average 0.21 dB and SSIM by 0.0058, all highly statistically significant (*p* < 0.01). Notably, the SSIM improvements of 0.0074 and 0.0087 on the IR700_test and DLS-NUC-100 test sets, underscoring the module’s ability to strengthen feature processing and refine denoising. Ultimately, the complete model combining the WTSA, MSGLU, and PHAM achieves optimal performance. It surpasses the BASIC baseline by average margins of 0.31 dB in PSNR and 0.0088 in SSIM, with all improvements highly statistically significant (*p* < 0.01). This superior performance highlights the PHAM module’s strength in spatial-channel feature fusion and detail enhancement, which effectively alleviates the issue of detail loss.

Furthermore, an ablation study on the placement of the PHAM is conducted. To this end, we designed two configurations: Encoder-PHAM, which inserts the module after the three WTTransformer modules (E1, E2, E3) in the encoder; Decoder-PHAM, where it is placed after the three WTTransformer modules (D1, D2, D3) in the Decoder. The experimental results are presented in Table 2.

The experimental results demonstrate that deploying the PHAM at any tested locations improves model performance, though the extent of improvement varies with the deployment position. Based on the average performance across the five test sets, the Encoder-PHAM configuration increases the PSNR by approximately 0.02 dB and the SSIM by 0.0006. The Decoder-PHAM configuration yields more notable gains, improving the PSNR by about 0.05 dB and the SSIM by 0.0015. Although both configurations provide certain performance gains, the improvements are not statistically significant compared to the baseline model (*p* > 0.05). In contrast, the proposed WTEIDM, which places PHAM between the encoder and decoder, achieves the most pronounced enhancement. It yields an average improvement of roughly 0.06 dB in PSNR and 0.0018 in SSIM, with both gains being statistically significant (*p* < 0.05).

These findings demonstrate that the PHAM effectively enhances feature refinement at various locations, yet its efficacy is closely tied to its structural position. In particular, when placed between the encoder and decoder, the module fully leverages its capability for cross-level information fusion and feature enhancement, thereby boosting the overall denoising performance of the model on infrared images.

To verify the effectiveness of configuring the 3 × 3 and 5 × 5 depth-separable convolutional kernel sizes in the MSGLU module, we conducted ablation experiments comparing different kernel combinations using WTSA + PHAM as the baseline model. Four size combinations were investigated: (1 × 1, 3 × 3), (3 × 3, 5 × 5), (5 × 5, 7 × 7), and (3 × 3, 7 × 7). The results are detailed in Table 3.

The experimental results indicate that the performance of MSGLU module is affected significantly by the kernel size combinations, with the proposed (3 × 3, 5 × 5) configuration achieving the most optimal performance improvement. Specifically, in the average performance evaluation across five test sets, the (1 × 1, 3 × 3) combination decreased PSNR by approximately 0.09 dB and SSIM by 0.0033 compared to the baseline, with statistically significant differences (*p* < 0.05). This performance decline can be attributed to the limited receptive field of the 1 × 1 kernel, which struggles to effectively extract spatial dependencies between pixels, thereby limiting feature representation capabilities. In contrast, the (5 × 5, 7 × 7) and (3 × 3, 7 × 7) combinations showed no statistically significant difference from the baseline (*p* > 0.1). While the 7 × 7 kernel expands the receptive field, it introduces excessive background noise and redundant information, increasing computational cost without providing discriminative feature enhancement. The proposed (3 × 3, 5 × 5) combination improved average PSNR and SSIM by approximately 0.06 dB and 0.0024, respectively, with highly statistically significant improvements (*p* < 0.01). In this configuration, the 3 × 3 kernel effectively captures local fine-grained structures (such as edges and textures), while the 5 × 5 kernel appropriately models global contextual information, thereby achieving an effective balance between detail preservation and high-level semantic modeling.

These results provide a rationale for selecting the combination of 3 × 3 and 5 × 5 convolution kernels in the MSGLU module. This configuration achieves an effective balance between feature extraction capability, detail retention, and computational efficiency, which is crucial for further improving the denoising performance of the WTEIDM.

### 3.5. Comparative Experiments

To comprehensively evaluate the performance of the proposed WTEIDM, we compare it against several state-of-the-art image denoising algorithms, including DnCNN [26], DRUNet [27], MemNet [28], MWCNN [29], and IDTransformer [30]. To ensure an objective and fair comparison, all methods are trained under an identical protocol on the IR700 dataset with Gaussian noise (σ = 15, 25, and 50) and subsequently tested on five infrared test sets (IR700_test, IR100, Flir, ESPOL FIR, and DLS-NUC-100). The statistical significance of all methods was calculated by comparing them with IDTransformer. The specific *p*-values and detailed results are summarized in Table 4.

As can be seen from the results, the proposed WTEIDM method achieves the best performance across all noise levels and test sets, significantly outperforming other competing algorithms. Compared to the suboptimal model IDTransformer, WTEIDM shows consistent improvements: at σ = 15, it provides average gains of 0.05 dB in PSNR and 0.0023 in SSIM. These performance advantages are statistically significant (*p* < 0.05) on most test sets; Under σ = 50, the performance advantage is even more substantial, with an average PSNR increase of 0.16 dB and an SSIM improvement of 0.0035, all of which are highly statistically significant (*p* < 0.02). Under σ = 25, the model’s PSNR improves by approximately 0.06 dB and SSIM by approximately 0.0014. Although these gains fall near the conventional significance threshold (*p* ≈ 0.05–0.06), its consistently positive trend indicates potential for further optimization, warranting deeper validation in larger or more complex scenarios. Notably, WTEIDM maintains a stable leading performance across all test sets, demonstrating strong generalization capability and robustness.

To comprehensively evaluate the computational complexity and storage requirements of each model, we calculated the number of parameters, floating-point operations (FLOPs), inference speed (FPS), and weights of all models under the training conditions of the IR700 dataset (noise level σ = 15). The results are summarized in Table 5.

From an efficiency perspective, the proposed WTEIDM maintains a compact architecture with moderate computation and run-time cost. Compared to the BASIC baseline, it achieves a lighter configuration and faster inference, indicating that the design improves restoration performance without increasing deployment burden. Moreover, relative to IDTransformer, WTEIDM introduces only minimal additional complexity while maintaining a comparable inference speed, suggesting that the performance gains are attained with limited extra cost.

In summary, the proposed WTEIDM consistently outperforms existing advanced methods in quantitative metrics, with statistically significant improvements that validate its effectiveness for infrared image denoising. Simultaneously, it achieves a favorable balance among denoising quality, computational efficiency, and resource consumption. This balance is attributable to the fact that each core component contributes to performance gains while incurring controlled overhead, rendering the overall model well-suited for practical deployment.

### 3.6. Visualization of Experimental Results and Analysis

**Figure 5:**
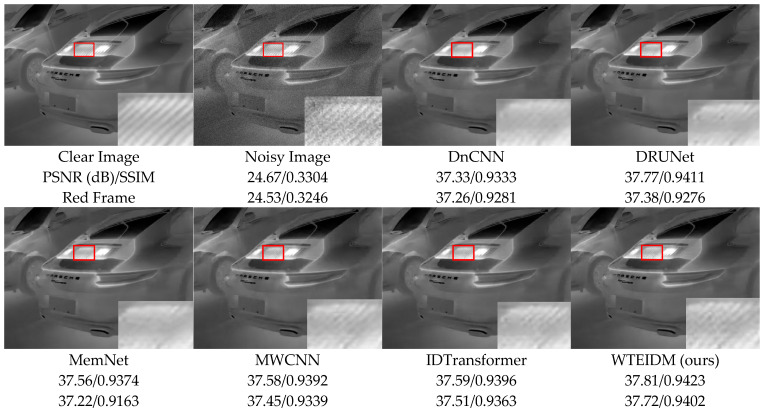
Comparisons of denoising results on image 37.png from the DLS-NUC-100 test dataset under noise level of σ = 15. Red boxes: magnified regions for detail comparison.

**Figure 6:**
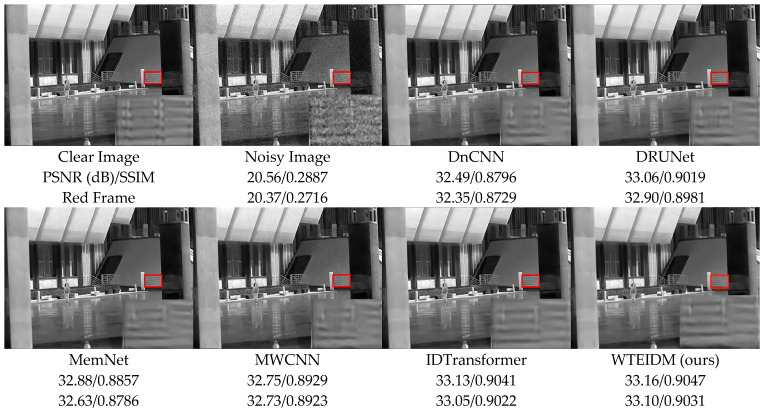
Comparison of denoising results on image 2.png from the IR700 test dataset under noise level of σ = 25. Red boxes: magnified regions for detail comparison.

**Figure 7:**
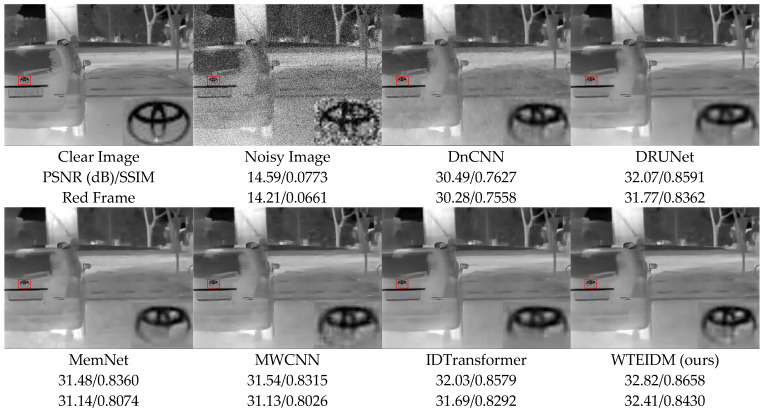
Comparison of denoising results on image 000982.png from the IR100 test dataset under noise level of σ = 50. Red boxes: magnified regions for detail comparison.

To validate the performance of the WTEIDM in infrared image denoising, we present visual comparisons of images with different noise levels from the test set, as shown in Figure 5, Figure 6 and Figure 7. The sub-figures are arranged as follows, from left to right: the original image, the noisy image, and the denoised results processed by DnCNN, DRUNet, MemNet, MWCNN, IDTransformer, and the proposed WTEIDM. A red box highlights a local region in each image, with a magnified view displayed in the lower-right corner for detailed comparison. The corresponding PSNR and SSIM values of both the full image and the red frame region are provided below each result to enable quantitative evaluation of local detail preservation.

Figure 5, Figure 6 and Figure 7 present visual comparisons of denoising results on different test images under noise levels of σ = 15, 25, 50, respectively. In Figure 5, the red box highlights the ventilation grille at the rear of the vehicle. While most comparative algorithms introduce shadows or blurring of details after denoising, WTEIDM effectively suppresses noise while better preserving the original structural features, demonstrating superior detail retention. In Figure 6, the red box marks a railing structure. Although IDTransformer achieves a reasonable balance between noise removal and feature preservation, WTEIDM outperforms it in both noise suppression and structural integrity, recovering railing details more completely. In Figure 7, the logo area shows that WTEIDM preserves contours and fine details more effectively than other methods.

Overall, the comprehensive results indicate that WTEIDM consistently restores key details under varying noise conditions, significantly enhancing the quality of infrared image denoising.

**Figure 8:**
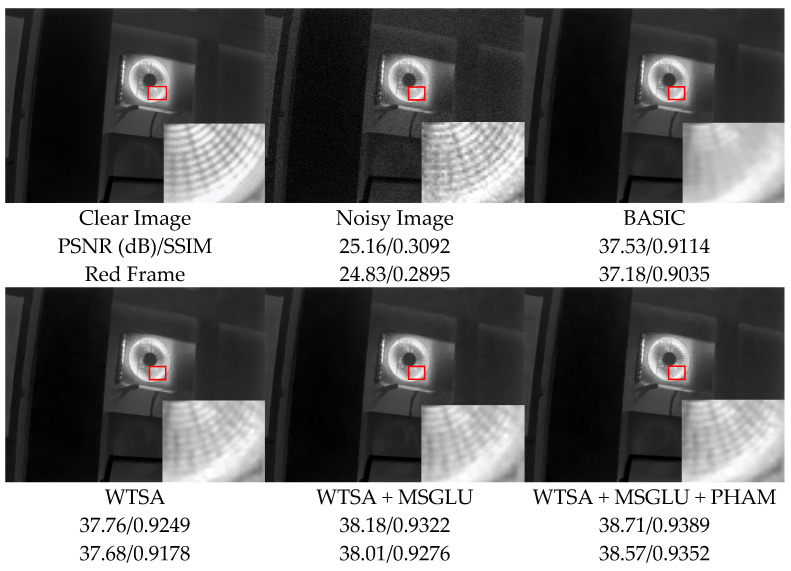
Comparison of denoising effects of the ablation experiment on image 34.png from the DLS-NUC-100 test dataset under noise level σ = 15. Red boxes: magnified regions for detail comparison.

We also visualized the ablation experiment results of sample “34.png” in the test dataset DLS-NUC-100 to visually verify the effectiveness of each core module, as shown in Figure 8. To highlight structural details, the red-boxed regions in each image are locally magnified for detailed comparison and analysis.

The visualization results show that the noisy image is severely affected by noise, and the structural texture information is significantly blurred. Although the BASIC model achieves a preliminary denoising effect, noise remains in some local areas, and edge details are not fully restored. In contrast, integrating the WTSA module significantly improves the image denoising quality, effectively enhancing the restoration of details and the clarity of structure, thus verifying the effectiveness of WTSA in separating noise and detail features. The introduction of the MSGLU module further optimized the noise reduction performance, resulting in clearer edge contours and textures, effectively alleviating the blurring of details caused by insufficient feature modulation. Ultimately, the complete model integrating the WTSA, MSGLU, and PHAM presented the best visual restoration effect, not only completely suppressing residual noise but also reconstructing the fine structural details in the image in a more natural and clear manner.

The above visualization results are consistent with the quantitative analysis conclusions in Section 3.4, jointly validating the progressive optimization mechanism of each core module: WTSA achieves the preliminary separation of noise and detail features, MSGLU further strengthens the extraction capability of multi-scale detail information, and PHAM facilitates cross-dimensional feature fusion between spatial and channel dimensions. Together, they enable a better balance between noise suppression and structural detail preservation.

**Figure 9:**
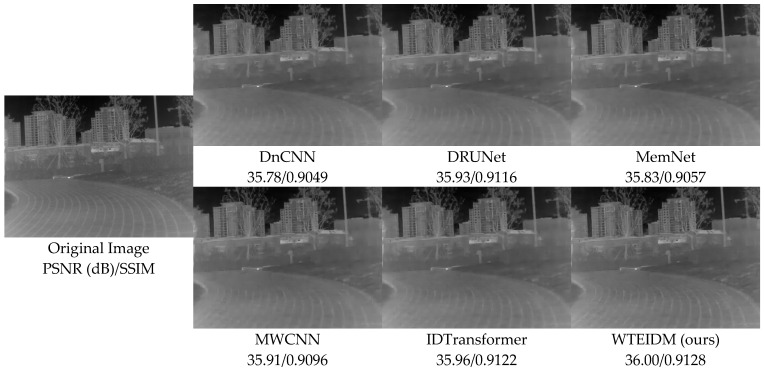
Comparison of denoising results various methods on image XDU228.png from the IRSTD-1k dataset.

**Figure 10:**
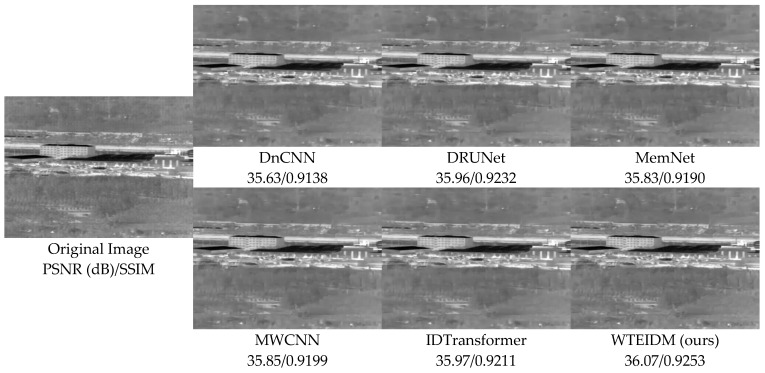
Comparison of denoising results of various methods on the image XDU1000.png in the IRSTD-1k dataset.

To validate the generalization ability and robustness of the proposed WTEIDM on real-world noisy infrared images, we conducted supplementary experiments on the public dataset IRSTD-1k [31]. Figure 9 and Figure 10, respectively, show the denoising results of different methods on the samples “XDU228.png” and “XDU1000.png” under real noise conditions. The experimental results demonstrate that even in real noise environments, WTEIDM still achieves the best performance in terms of both PSNR and SSIM metrics, and significantly outperforms other methods in both structural integrity and textural details preservation. Specifically, in Figure 9, WTEIDM exhibits outstanding performance in restoring runway stripes and ground textures. In Figure 10, the model can also better preserve the basic contours and local details of the wooded area. These results show that WTEIDM can still effectively achieve noise suppression and detail preservation in the face of mixed noise or structural noise interference that exist in real scenes, further verifying its good robustness and generalization ability in practical applications.

## 4. Limitation

Although WTEIDM achieves a favorable efficiency–performance trade-off, there is still room for improvement in model compactness and deployment cost. As summarized in Table 5, WTEIDM (18.79 M parameters and 14.20 G FLOPs) is notably lighter than the BASIC baseline, but it is still less compact than some lightweight CNN-based alternatives. Moreover, compared with IDTransformer, WTEIDM introduces additional computational overhead in terms of parameters and FLOPs, which may be a consideration under strict edge-device budgets. Furthermore, in a small number of challenging cases, the denoised results may exhibit slight over-smoothing or minor residual noise, indicating that detail preservation in complex scenes or regions could be further improved.

In future work, we will pursue more deployment-friendly designs by developing efficient attention and parameter-sharing strategies, along with implementation-level optimization, to further reduce parameters, FLOPs, and memory footprint while maintaining denoising quality. We will also explore model compression and acceleration techniques, such as pruning, quantization, and knowledge distillation, to enable real-time inference on resource-constrained infrared devices. To better handle challenging samples, we plan to incorporate structure-aware supervision (e.g., edge/gradient constraints) and refine multi-scale feature modulation, enabling more faithful preservation of weak textures and subtle thermal boundaries. Furthermore, we will extend training and evaluation to more realistic infrared degradations and sensor-dependent noise patterns, and investigate noise-aware adaptation to improve generalization in practical imaging scenarios.

## 5. Conclusions

This paper proposes a wavelet transform-based enhanced infrared denoising model (WTEIDM). The model incorporates a wavelet transform self-attention (WTSA) mechanism, which achieves effective separation of noise and detail components in the frequency domain and enhances the perception of key features. A multi-scale gated linear unit (MSGLU) is designed to improve the richness of feature representation and adaptability regulation capability through a multi-branch convolutional structure and gating mechanism. Additionally, a parallel hybrid attention module (PHAM) is introduced to further strengthen cross-dimensional feature fusion and detail retention through the complementarity interaction of spatial and channel attention paths. Extensive experiments are conducted on five infrared datasets under different noise levels. The results demonstrate that the proposed method achieves superior denoising performance in terms of PSNR and SSIM metrics compared to existing denoising methods.

## Figures and Tables

**Table 1:** Ablation experiment with Gaussian noise σ of 15 (mean ± SD; *p*-values).

Module	IR700_Test		IR100		Flir		ESPOL FIR		DLS-NUC-100	
	PSNR	SSIM	PSNR	SSIM	PSNR	SSIM	PSNR	SSIM	PSNR	SSIM
BASIC	34.74 ±0.04 (-)	0.8828 ±0.0015 (-)	37.65 ±0.05 (-)	0.9191 ±0.0012 (-)	35.79 ±0.06 (-)	0.9209 ±0.0014 (-)	36.13 ±0.04 (-)	0.8769 ±0.0013 (-)	36.16 ±0.05 (-)	0.8684 ±0.0016 (-)
WTSA	34.82 ±0.05 (0.038)	0.8862 ±0.0013 (0.032)	37.75 ±0.04 (0.035)	0.9210 ±0.0010 (0.031)	35.92 ±0.05 (0.039)	0.9242 ±0.0012 (0.034)	36.19 ±0.05 (0.047)	0.8780 ±0.0011 (0.043)	36.24 ±0.04 (0.041)	0.8713 ±0.0014 (0.037)
PHAM	34.79 ±0.04 (0.045)	0.8854 ±0.0014 (0.039)	37.71 ±0.05 (0.042)	0.9201 ±0.0011 (0.038)	35.89 ±0.06 (0.043)	0.9234 ±0.0013 (0.036)	36.18 ±0.04 (0.049)	0.8777 ±0.0012 (0.046)	36.21 ±0.05 (0.044)	0.8702 ±0.0015 (0.040)
WTSA + MSGLU	34.94 ±0.06 (0.009)	0.8902 ±0.0012 (0.008)	37.89 ±0.05 (0.007)	0.9241 ±0.0010 (0.006)	36.01 ±0.05 (0.008)	0.9263 ±0.0011 (0.007)	36.25 ±0.04 (0.012)	0.8793 ±0.0010 (0.011)	36.41 ±0.06 (0.007)	0.8771 ±0.0013 (0.006)
WTSA + PHAM	34.88 ±0.05 (0.014)	0.8878 ±0.0013 (0.012)	37.87 ±0.04 (0.011)	0.9235 ±0.0011 (0.010)	35.96 ±0.06 (0.013)	0.9250 ±0.0012 (0.011)	36.22 ±0.05 (0.018)	0.8786 ±0.0011 (0.016)	36.36 ±0.05 (0.012)	0.8751 ±0.0014 (0.010)
WTSA+ MSGLU+ PHAM	**35.05** **±0.06** **(0.003)**	**0.8939** **±0.0011** **(0.002)**	**37.96** **±0.05** **(0.002)**	**0.9258** **±0.0009** **(0.002)**	**36.14** **±0.05** **(0.003)**	**0.9294** **±0.0010** **(0.002)**	**36.38** **±0.04** **(0.004)**	**0.8816** **±0.0009** **(0.003)**	**36.50** **±0.06** **(0.002)**	**0.8812** **±0.0012** **(0.002)**

**Table 2:** The ablation experiment results of PHAM at different positions (mean ± SD; *p*-values vs. BASIC baseline in parentheses).

Module	IR700_Test		IR100		Flir		ESPOL FIR		DLS-NUC-100	
	PSNR	SSIM	PSNR	SSIM	PSNR	SSIM	PSNR	SSIM	PSNR	SSIM
BASIC	34.74 ±0.04 (-)	0.8828 ±0.0015 (-)	37.65 ±0.05 (-)	0.9191 ±0.0012 (-)	35.79 ±0.06 (-)	0.9209 ±0.0014 (-)	36.13 ±0.04 (-)	0.8769 ±0.0013 (-)	36.16 ±0.05 (-)	0.8684 ±0.0016 (-)
Encoder-PHAM	34.76 ±0.04 (0.189)	0.8834 ±0.0014 (0.213)	37.66 ±0.05 (0.207)	0.9193 ±0.0011 (0.225)	35.84 ±0.06 (0.164)	0.9222 ±0.0013 (0.178)	36.15 ±0.04 (0.192)	0.8772 ±0.0012 (0.205)	36.18 ±0.05 (0.175)	0.8691 ±0.0015 (0.188)
Decoder-PHAM	34.78 ±0.04 (0.063)	0.8850 ±0.0013 (0.071)	37.69 ±0.05 (0.067)	0.9198 ±0.0011 (0.074)	35.88 ±0.06 (0.059)	0.9232 ±0.0012 (0.065)	36.18 ±0.04 (0.068)	0.8777 ±0.0012 (0.072)	36.20 ±0.05 (0.064)	0.8698 ±0.0015 (0.070)
PHAM	**34.79** **±0.04** **(0.045)**	**0.8854** **±0.0013** **(0.039)**	**37.71** **±0.05** **(0.042)**	**0.9201** **±0.0011** **(0.038)**	**35.90** **±0.06** **(0.043)**	**0.9237** **±0.0012** **(0.036)**	**36.18** **±0.04** **(0.049)**	**0.8777** **±0.0012** **(0.046)**	**36.21** **±0.05** **(0.044)**	**0.8702** **±0.0015** **(0.040)**

**Table 3:** The ablation experiment results of Kernel Size Configuration in the MSGLU Module (mean ± SD; *p*-values).

Kernel Size Combination	IR700_Test		IR100		Flir		ESPOL FIR		DLS-NUC-100	
	PSNR	SSIM	PSNR	SSIM	PSNR	SSIM	PSNR	SSIM	PSNR	SSIM
Baseline (WTSA + PHAM)	34.88 ±0.05 (-)	0.8878 ±0.0013 (-)	37.87 ±0.04 (-)	0.9235 ±0.0011 (-)	35.96 ±0.06 (-)	0.9250 ±0.0012 (-)	36.22 ±0.05 (-)	0.8786 ±0.0011 (-)	36.36 ±0.05 (-)	0.8751 ±0.0014 (-)
(1 × 1, 3 × 3)	34.79 ±0.04 (0.028)	0.8845 ±0.0012 (0.025)	37.76 ±0.05 (0.031)	0.9208 ±0.0010 (0.029)	35.85 ±0.05 (0.033)	0.9221 ±0.0011 (0.030)	36.15 ±0.04 (0.036)	0.8764 ±0.0010 (0.032)	36.27 ±0.04 (0.034)	0.8723 ±0.0013 (0.031)
(5 × 5, 7 × 7)	34.85 ±0.05 (0.156)	0.8869 ±0.0013 (0.162)	37.83 ±0.04 (0.149)	0.9227 ±0.0011 (0.155)	35.93 ±0.06 (0.151)	0.9242 ±0.0012 (0.158)	36.20 ±0.05 (0.163)	0.8781 ±0.0011 (0.157)	36.33 ±0.05 (0.153)	0.8743 ±0.0014 (0.160)
(3 × 3, 7 × 7)	34.86 ±0.05 (0.112)	0.8873 ±0.0013 (0.121)	37.84 ±0.04 (0.108)	0.9230 ±0.0011 (0.115)	35.94 ±0.06 (0.113)	0.9245 ±0.0012 (0.119)	36.21 ±0.05 (0.124)	0.8783 ±0.0011 (0.118)	36.34 ±0.05 (0.109)	0.8746 ±0.0014 (0.116)
(3 × 3, 5 × 5) (ours)	**34.94** **±0.06** **(0.009)**	**0.8902** **±0.0012** **(0.008)**	**37.89** **±0.05** **(0.007)**	**0.9241** **±0.0010** **(0.006)**	**36.01** **±0.05** **(0.008)**	**0.9263** **±0.0011** **(0.007)**	**36.25** **±0.04** **(0.012)**	**0.8793** **±0.0010** **(0.011)**	**36.41** **±0.06** **(0.007)**	**0.8771** **±0.0013** **(0.006)**

**Table 4:** The comparative experimental results of different methods (mean ± SD; *p*-values).

Module	σ	IR700_Test		IR100		Flir		ESPOL FIR		DLS-NUC-100	
		PSNR	SSIM	PSNR	SSIM	PSNR	SSIM	PSNR	SSIM	PSNR	SSIM
DnCNN [26]	15	34.71 ±0.05 (<0.001)	0.8817 ±0.0016 (<0.001)	37.48 ±0.06 (<0.001)	0.9153 ±0.0014 (<0.001)	35.72 ±0.07 (<0.001)	0.9198 ±0.0015 (<0.001)	36.11 ±0.05 (<0.001)	0.8760 ±0.0014 (<0.001)	36.13 ±0.06 (<0.001)	0.8674 ±0.0017 (<0.001)
DRUNet [27]		34.98 ±0.06 (0.023)	0.8914 ±0.0013 (0.037)	37.87 ±0.05 (0.032)	0.9238 ±0.0011 (0.039)	36.05 ±0.06 (0.042)	0.9272 ±0.0012 (0.045)	36.35 ±0.05 (0.048)	0.8810 ±0.0010 (0.051)	36.42 ±0.06 (0.044)	0.8785 ±0.0014 (0.047)
MemNet [28]		34.88 ±0.05 (<0.001)	0.8852 ±0.0014 (<0.001)	37.63 ±0.06 (<0.001)	0.9200 ±0.0013 (<0.001)	35.88 ±0.07 (<0.001)	0.9223 ±0.0014 (<0.001)	36.23 ±0.05 (<0.001)	0.8772 ±0.0013 (<0.001)	36.28 ±0.06 (<0.001)	0.8737 ±0.0016 (<0.001)
MWCNN [29]		34.97 ±0.06 (0.029)	0.8901 ±0.0013 (0.097)	37.85 ±0.05 (0.092)	0.9258 ±0.0010 (0.095)	35.99 ±0.06 (0.026)	0.9254 ±0.0013 (0.028)	36.30 ±0.05 (0.021)	0.8786 ±0.0011 (0.023)	36.42 ±0.06 (0.041)	0.8788 ±0.0014 (0.043)
IDTransformer [30]		35.00 ±0.06 (-)	0.8925 ±0.0012 (-)	37.89 ±0.05 (-)	0.9249 ±0.0010 (-)	36.07 ±0.06 (-)	0.9281 ±0.0012 (-)	36.36 ±0.05 (-)	0.8812 ±0.0010 (-)	36.44 ±0.06 (-)	0.8796 ±0.0014 (-)
WTEIDM (ours)		**35.05** **±0.06** **(0.039)**	**0.8939** **±0.0011** **(0.043)**	**37.96** **±0.05** **(0.041)**	**0.9258** **±0.0009** **(0.044)**	**36.14** **±0.06** **(0.035)**	**0.9294** **±0.0011** **(0.037)**	**36.38** **±0.05** **(0.053)**	**0.8816** **±0.0009** **(0.056)**	**36.50** **±0.06** **(0.038)**	**0.8812** **±0.0012** **(0.040)**
DnCNN [26]	25	32.57 ±0.07 (<0.001)	0.8402 ±0.0018 (<0.001)	35.25 ±0.08 (<0.001)	0.8912 ±0.0016 (<0.001)	33.26 ±0.08 (<0.001)	0.8772 ±0.0017 (<0.001)	34.25 ±0.07 (<0.001)	0.8373 ±0.0016 (<0.001)	34.18 ±0.07 (<0.001)	0.8378 ±0.0018 (<0.001)
DRUNet [27]		32.99 ±0.07 (0.028)	0.8596 ±0.0016 (0.032)	36.15 ±0.07 (0.031)	0.9083 ±0.0014 (0.033)	33.83 ±0.08 (0.029)	0.8964 ±0.0015 (0.030)	34.89 ±0.07 (0.034)	0.8598 ±0.0015 (0.035)	34.94 ±0.07 (0.036)	0.8608 ±0.0017 (0.037)
MemNet [28]		32.89 ±0.07 (<0.001)	0.8517 ±0.0017 (<0.001)	35.72 ±0.08 (<0.001)	0.8989 ±0.0015 (<0.001)	33.39 ±0.08 (<0.001)	0.8852 ±0.0016 (<0.001)	34.61 ±0.07 (<0.001)	0.8480 ±0.0016 (<0.001)	34.71 ±0.07 (<0.001)	0.8515 ±0.0017 (<0.001)
MWCNN [29]		32.78 ±0.07 (<0.001)	0.8528 ±0.0017 (0.012)	35.80 ±0.08 (0.011)	0.9057 ±0.0015 (0.010)	33.56 ±0.08 (0.013)	0.8891 ±0.0016 (0.014)	34.68 ±0.07 (0.010)	0.8536 ±0.0016 (0.009)	34.61 ±0.07 (0.008)	0.8540 ±0.0017 (0.007)
IDTransformer [30]		33.06 ±0.07 (-)	0.8605 ±0.0016 (-)	36.10 ±0.07 (-)	0.9071 ±0.0014 (-)	33.90 ±0.08 (-)	0.8975 ±0.0015 (-)	34.96 ±0.07 (-)	0.8606 ±0.0015 (-)	34.91 ±0.07 (-)	0.8600 ±0.0017 (-)
WTEIDM (ours)		**33.10** **±0.07** **(0.049)**	**0.8622** **±0.0015** **(0.053)**	**36.19** **±0.07** **(0.051)**	**0.9096** **±0.0013** **(0.054)**	**33.92** **±0.08** **(0.059)**	**0.8979** **±0.0015** **(0.061)**	**35.02** **±0.07** **(0.057)**	**0.8612** **±0.0015** **(0.058)**	**35.02** **±0.07** **(0.052)**	**0.8619** **±0.0016** **(0.054)**
DnCNN [26]	50	28.98 ±0.09 (<0.001)	0.7250 ±0.0024 (<0.001)	31.40 ±0.10 (<0.001)	0.7816 ±0.0021 (<0.001)	29.54 ±0.10 (<0.001)	0.7499 ±0.0023 (<0.001)	31.16 ±0.09 (<0.001)	0.7424 ±0.0022 (<0.001)	30.78 ±0.09 (<0.001)	0.7370 ±0.0024 (<0.001)
DRUNet [27]		30.07 ±0.09 (0.014)	0.8034 ±0.0020 (0.017)	33.38 ±0.10 (0.016)	0.8842 ±0.0018 (0.018)	30.83 ±0.10 (0.019)	0.8400 ±0.0020 (0.020)	32.81 ±0.09 (0.021)	0.8294 ±0.0019 (0.022)	32.53 ±0.09 (0.020)	0.8298 ±0.0021 (0.021)
MemNet [28]		29.68 ±0.09 (<0.001)	0.7778 ±0.0022 (<0.001)	32.04 ±0.10 (<0.001)	0.8504 ±0.0020 (<0.001)	29.66 ±0.10 (<0.001)	0.8073 ±0.0022 (<0.001)	31.95 ±0.09 (<0.001)	0.7963 ±0.0021 (<0.001)	31.95 ±0.09 (<0.001)	0.8033 ±0.0023 (<0.001)
MWCNN [29]		29.65 ±0.09 (<0.001)	0.7790 ±0.0022 (<0.001)	32.75 ±0.10 (<0.001)	0.8551 ±0.0019 (<0.001)	30.41 ±0.10 (<0.001)	0.8149 ±0.0021 (<0.001)	32.18 ±0.09 (<0.001)	0.7977 ±0.0021 (<0.001)	31.92 ±0.09 (<0.001)	0.8008 ±0.0023 (<0.001)
IDTransformer [30]		30.17 ±0.09 (-)	0.8068 ±0.0020 (-)	33.24 ±0.10 (-)	0.8803 ±0.0018 (-)	30.86 ±0.10 (-)	0.8416 ±0.0020 (-)	32.71 ±0.09 (-)	0.8262 ±0.0019 (-)	32.48 ±0.09 (-)	0.8281 ±0.0021 (-)
WTEIDM (ours)		**30.32** **±0.09** **(0.011)**	**0.8103** **±0.0019** **(0.012)**	**33.49** **±0.10** **(0.013)**	**0.8846** **±0.0017** **(0.014)**	**31.01** **±0.10** **(0.015)**	**0.8446** **±0.0019** **(0.016)**	**32.84** **±0.09** **(0.017)**	**0.8297** **±0.0018** **(0.018)**	**32.62** **±0.09** **(0.014)**	**0.8311** **±0.0020** **(0.015)**

**Table 5:** A comprehensive comparison of different methods in terms of computational efficiency and resource consumption.

Module	Parameters (M)	FLOPs (G)	FPS	Weights Size (MB)
BASIC	24.51	18.72	88.5	93.6
DnCNN [26]	0.55	1.27	247.6	2.2
DRUNet [27]	32.63	25.47	62.4	124.0
MemNet [28]	2.91	7.68	185.0	11.3
MWCNN [29]	16.15	12.85	125.0	61.6
IDTransformer [30]	18.58	14.05	113.8	71.1
WTEIDM (ours)	18.79	14.20	111.5	72.0

## Data Availability

No new data were created or analyzed in this study.

## References

[1] Yin Z., Liu S., Tong X. (2023). Infrared image denoising algorithm based on domain adaptation. J. Laser Infrared.

[2] Ou W., Wan L. (2023). Infrared image denoising based on double density complex wavelet and coefficient correlation. J. Opt. Tech..

[3] Han H. (2015). Improved non-local means filtering algorithm for infrared images in NSCT domain. J. Infrared Technol..

[4] Gu D. (2024). Bilateral weighted median filter for removing impulse noise in infrared image. J. Chin. J. Sens. Actuators.

[5] Hao J., Du Y., Wang S., Ren J. (2024). Infrared image enhancement algorithm based on wavelet transform and improved bilateral filtering. J. Infrared Technol..

[6] Zhai P., Wang P. (2021). Application of adaptive Wiener filter in molten steel infrared image denoising. J. Infrared Technol..

[7] Liu G., Gong Y., Zhang H., Liang H. (2024). Infrared spectrum denoising algorithm based on wavelet transform optimization EEMD combined with SG. J. Infrared Technol..

[8] Wu J., Niu H., Zhang H., Xu J. (2018). A hybrid Fourier-wavelet method for infrared image denoising of cable porcelain terminal based on wavelet coefficient GSM model. J. Electr. Meas. Instrum..

[9] Buades A., Coll B., Morel J. (2011). Non-local means denoising. J. Image Process. Line.

[10] Dabov K., Foi A., Katkovnik V., Egiazarian K. (2007). Image denoising by sparse 3-D transform-domain collaborative filtering. J. IEEE Trans. Image Process..

[11] Arnim G., Aditi P., Aishwarya J., Satya N.T., Sachin C., Praful H., Akshay D., Santosh V., Subrahamanyam M. Pureformer: Transformer-Based Image Denoising. Proceedings of the IEEE/CVF Conference on Computer Vision and Pattern Recognition (CVPR 2025).

[12] Li J., Zhang Z., Zuo W. (2025). Rethinking Transformer-Based Blind-Spot Network for Self-Supervised Image Denoising. J. Proc. AAAI Conf. Artif. Intell..

[13] Jin Z., Qiu Y., Zhang K., Li H., Luo W. (2025). MB-TaylorFormer V2: Improved Multi-Branch Linear Transformer Expanded by Taylor Formula for Image Restoration. J. IEEE Trans. Pattern Anal. Mach. Intell..

[14] Zhang K., Li R., Yu Y., Luo W., Li C. (2021). Deep Dense Multi-Scale Network for Snow Removal Using Semantic and Depth Priors. J. IEEE Trans. Image Process..

[15] Li Z., Luo S., Chen M., Wu H., Wang T., Cheng L. (2021). Infrared thermal imaging denoising method based on second-order channel attention mechanism. J. Infrared Phys. Technol..

[16] Zhang Z., Zheng W., Ma Z., Yin L., Xie M., Wu Y. (2021). Infrared star image denoising using regions with deep reinforcement learning. J. Infrared Phys. Technol..

[17] Hu X., Luo S., He C., Wu W., Wu H. (2023). Infrared thermal image denoising with symmetric multi-scale sampling network. J. Infrared Phys. Technol..

[18] Yang P., Wu H., Cheng L., Luo S. (2023). Infrared image denoising via adversarial learning with multi-level feature attention network. J. Infrared Phys. Technol..

[19] Wu W., Dong X., Li R., Chen H., Cheng L. (2024). SwinDenoising: A Local and Global Feature Fusion Algorithm for Infrared Image Denoising. J. Math..

[20] Vaswani A., Shazeer N., Parmar N., Uszkoreit J., Jones L., Gomez A.N., Kaiser Ł., Polosukhin I. Attention is all you need. Proceedings of the 31st Conference on Neural Information Processing Systems (NIPS 2017).

[21] Zou Y., Zhang L., Liu C., Wang B., Hu Y., Chen Q. (2021). Super-resolution reconstruction of infrared images based on a convolutional neural network with skip connections. J. Opt. Lasers Eng..

[22] Huang Y., Jiang Z., Lan R., Zhang S., Pi K. (2021). Infrared image super-resolution via transfer learning and PSRGAN. J. IEEE Signal Process. Lett..

[23] Rivadeneira R.E., Sappa A.D., Vintimilla B.X. Thermal image super-resolution: A novel architecture and dataset. Proceedings of the 15th International Joint Conference on Computer Vision, Imaging and Computer Graphics Theory and Application (VISIGRAPP 2020).

[24] Rivadeneira R.E., Suárez P.L., Sappa A.D., Vintimilla B.X. Thermal Image SuperResolution Through Deep Convolutional Neural Network. Proceedings of the International Conference on Image Analysis and Recognition (ICIAR 2019).

[25] He Z., Cao Y., Dong Y., Yang J., Cao Y., Tisse C. (2018). Single-image-based nonuniformity correction of uncooled long-wave infrared detectors: A deep-learning approach. J. Appl. Opt..

[26] Zhang K., Zuo W., Chen Y., Meng D., Zhang L. (2017). Beyond a gaussian denoiser: Residual learning of deep cnn for image denoising. J. IEEE Trans. Image Process..

[27] Zhang K., Li Y., Zuo W., Zhang L., Gool L.V., Timofte R. (2021). Plug-and-play image restoration with deep denoiser prior. J. IEEE Trans. Pattern Anal. Mach. Intell..

[28] Tai Y., Yang J., Liu X., Xu C. Memnet: A persistent memory network for image restoration. Proceedings of the IEEE International Conference on Computer Vision (ICCV 2017).

[29] Liu P., Zhang H., Zhang K., Lin L., Zuo W. Multi-level wavelet-CNN for image restoration. Proceedings of the IEEE Conference on Computer Vision and Pattern Recognition Workshops (CVPR 2018).

[30] Shen Z., Qin F., Ge R., Wang C., Zhang K., Huang J. (2025). IDTransformer: Infrared image denoising method based on convolutional transposed self-attention. Alex. Eng. J..

[31] Zhang M., Zhang R., Yang Y., Bai H., Zhang J., Guo J. ISNet: Shape Matters for Infrared Small Target Detection. Proceedings of the IEEE/CVF Conference on Computer Vision and Pattern Recognition (CVPR 2022).

